# T cell morphodynamics reveal periodic shape oscillations in three-dimensional migration

**DOI:** 10.1098/rsif.2022.0081

**Published:** 2022-05-11

**Authors:** Henry Cavanagh, Daryan Kempe, Jessica K. Mazalo, Maté Biro, Robert G. Endres

**Affiliations:** ^1^ Imperial College London, Centre for Integrative Systems Biology and Bioinformatics, London SW7 2BU, UK; ^2^ EMBL Australia, Single Molecule Science Node, School of Medical Sciences, The University of New South Wales, Sydney, NSW 2052, Australia

**Keywords:** T cells, morphodynamics, lattice light-sheet microscope, physics of behaviour, shape analysis

## Abstract

T cells use sophisticated shape dynamics (morphodynamics) to migrate towards and neutralize infected and cancerous cells. However, there is limited quantitative understanding of the migration process in three-dimensional extracellular matrices (ECMs) and across timescales. Here, we leveraged recent advances in lattice light-sheet microscopy to quantitatively explore the three-dimensional morphodynamics of migrating T cells at high spatio-temporal resolution. We first developed a new shape descriptor based on spherical harmonics, incorporating key polarization information of the uropod. We found that the shape space of T cells is low-dimensional. At the behavioural level, run-and-stop migration modes emerge at approximately 150 s, and we mapped the morphodynamic composition of each mode using multiscale wavelet analysis, finding ‘stereotyped’ motifs. Focusing on the run mode, we found morphodynamics oscillating periodically (every approx. 100 s) that can be broken down into a biphasic process: front-widening with retraction of the uropod, followed by a rearward surface motion and forward extension, where intercalation with the ECM in both of these steps likely facilitates forward motion. Further application of these methods may enable the comparison of T cell migration across different conditions (e.g. differentiation, activation, tissues and drug treatments) and improve the precision of immunotherapeutic development.

## 1. Introduction

**Figure 1.**
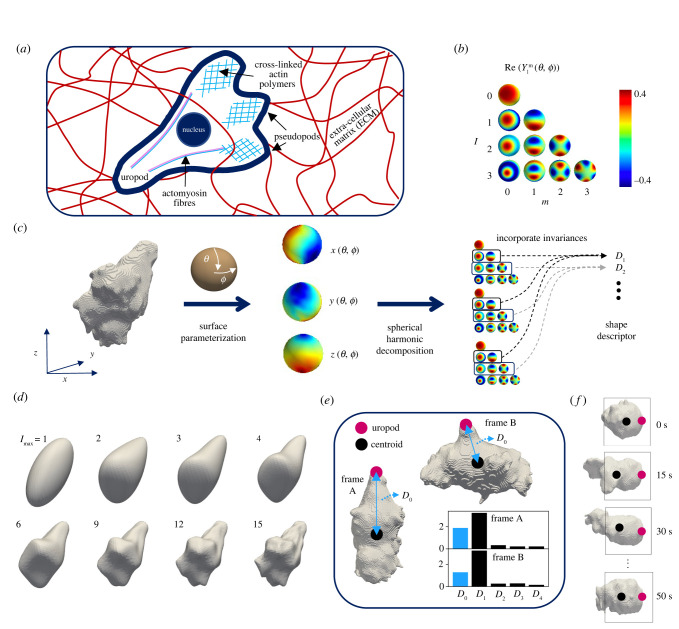
T cell shape can be quantified by spherical harmonic descriptors in three dimensions. (*a*) Schematic of a T cell employing an amoeboid migration strategy to navigate through the extracellular matrix (ECM) in three dimensions. Actin polymerization at the front results in the formation of pseudopods, and a complex of actomyosin at the rear forms the uropod, important for stability and generating contractile forces. (*b*) Complex spherical harmonic functions, $Y_{l}^{m}(\theta ,\phi )$ where *l* is the function degree (related to frequency) and *m* is the order (rotations at each degree; real parts shown for *m* ≥ 0), form a basis on the surface of a sphere. (*c*) Cartesian coordinates of the cell surface, {*x*, *y*, *z*}, are mapped to the surface of a sphere, as parameterized by polar coordinates {*θ*, *ϕ*}. The three resulting functions {*x*(*θ*, *ϕ*), *y*(*θ*, *ϕ*), *z*(*θ*, *ϕ*)} are decomposed in terms of the spherical harmonic functions and transformed to be translation, scale and rotation invariant. This yields the final shape representation, *D*_*l*_, based on the harmonics at each energy level, *l*, with the exclusion of *l* = 0 giving the translation invariance. (*d*) Truncation of the representation at different degrees of *l* leads to different levels of smoothing, with *l* = 1 describing the ellipsoid part of the shape. (*e*) An additional descriptor, *D*_0_, for accounting for cell orientation, with the landmark-like smooth uropod at the rear and dynamic protrusions at the leading edge. Without this additional variable, the two cells shown have very similar descriptors. The standard deviation of *D*_0_ across all datasets is 0.31, and the standard deviations of the remaining *D*_*l*_ are all lower. (*f* ) For cases where the uropod vanishes, the landmark-like rear can still be identified by its smoothness and stationarity, compared with the dynamic leading edge, as shown in the example. For simplicity, we refer to this region at the rear as the uropod for all frames.

Shape changes (morphodynamics) are one of the principal mechanisms through which individual cells interact with their environment [1,2]. These dynamics arise from the interplay between a multitude of molecules and complex signalling pathways that often organize with emergent simplicity to carry out critical cellular functions, including division and migration. T cells, specialized cells of the adaptive immune system, are highly dependent on global morphodynamics to squeeze through gaps in the extracellular matrix (ECM), in contrast to the ECM-degrading strategies other cells use (e.g. tumour cells). Despite plasticity for adjusting the mode of migration to environmental conditions, the migration of T cells is often characterized as amoeboid: fast (up to 25 μm min^−1^ [3]), with low adhesion and polarized morphologies arising due to the segregation of different cytoskeletal networks to specific subcellular compartments [4]. In this mode of locomotion, dynamic F-actin forms pseudopods at the leading edge and an actomyosin-rich uropod at the rear generates contractile forces [5] (see figure 1*a* for a schematic). However, this canonical migration mechanism is not fixed and T cells adapt their motility to their immediate environment.

T cells are thought to toggle between exploration and exploitation states, balancing surface receptor cues for interacting with antigen-presenting or target cells (stop) with chemokine-driven or purely exploratory searches (run) [6]. The specific morphodynamics and force-generating mechanisms behind these states are not well understood, in part due to their large variety and adaptability in different environments [7]. Proposed methods for propulsion include leading edge extension and intercalation with the ECM (using either low-adhesion integrin connections or surface texture for friction), followed by contraction of the uropod for small pore sizes [7,8]. In addition to creating friction for moving forward, the rearward flow of actin waves from the leading edge may connect with the ECM like a paddle [9,10]. However, the extent to which these methods are used in complex three-dimensional ECM environments, and their precise organization, are far from well-characterized.

Accurate characterization is important as dysregulation of T cell migration processes can be highly deleterious. T cells differentiate into different effector states. For instance, antigen-specific CD4^+^ ‘helper’ T cells amplify the immune response, while CD8^+^ ‘cytotoxic’ T cells seek out and neutralize infected or cancerous cells [11]. Inadequate migration leaves infected and cancerous cells free to proliferate, while over-stimulation can cause inflammation-based diseases like asthma and arthritis [12]. While there are exciting immunotherapeutic avenues manipulating the migration process, these have so far disappointed [13]. With quantitative representations of T cell morphodynamics, their statistics can be interpreted with high precision and compared across conditions for potentially improved immunotherapeutic development, and mechanistic models can be developed [14–16].

One of the main challenges for analysing morphodynamics is that cells do not have obvious landmarks (e.g. legs, eyes, wings of animals), and so the important degrees of freedom must be inferred from the data itself. Where there is important landmark-like information (e.g. polarization that can manifest as subtle morphological features), this is typically diffuse rather than precisely locatable, which further complicates quantification. Current methods therefore do not explicitly include this information. Two-dimensional cell morphologies are often quantified using Fourier descriptors. This method decomposes the cell outline coordinates as functions of rotation around the centroid in terms of Fourier coefficients, which then represent the morphology. This approach has revealed that amoeboid migrating cells in two dimensions, including epithelial keratocytes and *Dictyostelium* amoebae [14,15], explore only a small subspace of the shapes that might be thought possible from qualitative inspection (i.e. low-dimensionality of morphology). Furthermore, the morphodynamics within this space are composed primarily of frequently used, or ‘stereotyped’, motifs (i.e. low-dimensionality of morphodynamics).

Imaging of three-dimensional cell dynamics at sufficiently high spatio-temporal resolution has only recently become available through lattice light-sheet microscopy (LLSM) [17]. Whether T cells navigating complex three-dimensional ECM environments similarly have low-dimensional morphology and morphodynamics remains to be understood. Such questions in three-dimensional necessitate automated analysis even more than in two dimensions, both because such datasets are inherently harder to visualize and interpret, and because three-dimensional environments typically induce a richer variety of morphodynamics [18]. Spherical harmonic descriptors (SPHARM), a three-dimensional analogue of Fourier descriptors, are a promising method for quantifying three-dimensional cell shape and connecting with motion [19]. However, the representations are typically too uninterpretable for exploring morphodynamics with high precision, and their use so far is primarily limited to classification or the detection of established shape changes [20,21]. It therefore remains an open question how best to quantify three-dimensional cell shapes without clear landmarks and interpret high spatio-temporal dynamics.

Here, we sought to combine LLSM [17] with quantitative image analysis to explore the three-dimensional morphodynamics of cytotoxic T cells migrating in the absence of chemoattractant cues through three-dimensional collagen matrices [22]. We first created a new compact shape descriptor, based on SPHARM, but better connected to key polarization information than current approaches. We found that T cells explore a low-dimensional morphological space, and that run-and-stop migration emerges at long timescales. We explored the morphodynamic compositions of these two modes using multiscale wavelet analysis, previously used to explore the structure of fruit fly behaviour [23,24], uncovering a global set of largely discrete stereotyped motifs. Focusing ultimately on the run mode, due to its key role in active translocation and polarized morphologies that are well-suited for analysis with our descriptor, we found that periodically oscillating morphodynamics (every approx. 100 s) sustain forward motion. These can be understood as a biphasic process integrating previously hypothesized propulsion mechanisms [9,10], namely: front-widening and retraction of the uropod (rear moves forward) and rearward surface motion with forward extension (front moves forward).

## 2. Results

### 2.1. T cell shape is low-dimensional

We imaged primary mouse effector CD8^+^ cytotoxic T cells in three-dimensional collagen matrices without chemical cues, with a lattice light-sheet microscope (LLSM) [17] at spatial resolutions of 0.145, 0.145, 0.4 μm and temporal resolution of approximately 2 −5 s (see §4 for details on the imaging and pre-processing and electronic supplementary material, figure S1 for a representative snapshot and three-dimensional trajectories). Spherical harmonics (figure 1*b*) can be used to quantify three-dimensional cell shapes, as shown in figure 1*c* [20,21,25,26]. The spherical harmonic functions, $Y_{l}^{m}(\theta ,\phi )$, form a basis over the sphere, where *l* is the function degree (related to frequency) and *m* is the order (rotations at each degree). The full approach is detailed in §4 and summarized here. The Cartesian coordinates describing the cell surface are each mapped to a sphere, so as polar coordinates {*θ*, *ϕ*} move over the sphere surfaces, the cell surface is traced out in object space. Analogous to a Fourier decomposition, the functions describing the cell surface can be decomposed into a set of spherical harmonic coefficients, $c_{l,i}^{m}$ with *i* ∈ {*x*, *y*, *z*}. The *l* = 0 coefficients describe the centroid location, the *l* = 1 coefficients describe the ellipsoid part of the shapes, and so on, with increasing levels of detail. Truncation of the representation at a certain *l*_max_ therefore leads to a representation of a smoothed version of the original morphology, where higher-frequency features are filtered out (figure 1*d*). Translation invariance is achieved by omitting the *l* = 0 coefficient, scale invariance is achieved by dividing all coefficients by *V*^−(1/3)^ where *V* is the volume [27], and rotational invariance is achieved by transforming to a new representation, {*D*_*l*_}_*l*>0_, with

2.1 $$D_{l}=\sum_{i\in (x,y,z)}\sum_{m=0}^{l}c_{l,i}^{m}c_{l,i}^{m*},$$

analogous to how rotational invariance can be achieved by extracting the power spectrum from Fourier descriptors of two-dimensional cell shapes [15]. There are two key problems with the descriptor in its current form, and we made two modifications to remedy these.

First, the coefficients are not linearly related to the spatial extent of different features. We therefore took the square root of each element, i.e. $\left\{D_{l}\}\to \{D_{l}^{1/2}\right\}$, which yields a descriptor more representative than the power spectrum [28]. Without this operation, almost all variance is contained in the first (ellipsoid) coefficient. Second, we added an element to the shape representation to capture key polarization information lost in a purely global shape representation. At the cell rear is the uropod, a smooth round appendage that stabilizes the cell and generates contractile forces, and at the leading edge emerge dynamic, higher-frequency protrusions. The cells in frames A and B in figure 1*e* have very similar descriptors under a regular spherical harmonic representation, reflecting the similarity of their ellipsoid components, but this misses the polarization conveyed in subtler features. We therefore added an extra descriptor, linearly related to the distance between the uropod and centroid, *D*_0_ (see §4 for the full expression). The standard deviation of *D*_0_ across all datasets is 0.31, and the standard deviations of the remaining *D*_*l*_ are all lower, showing that frames A and B in figure 1*e* are approximately two standard deviations apart along the *D*_0_ dimension. While most cells have a well-defined uropod that can be readily identified (e.g. frames A and B in figure 1*e*), some can exhibit more spherical shapes, as shown in figure 1*f*. However, even for these cells, there is still an identifiable smooth rear opposite a dynamic leading edge, and temporal information can reveal where the uropod transiently forms. For simplicity, we refer to this region at the rear as the uropod for all frames. The ultimate representation of T cell shape is therefore ${\left\{D_{l}\right\}}_{l=0}^{l_{\max}=15}$, with *D*_0_ as described above and *D*_*l*_ for *l* > 0 the square root of the expression in equation (2.1).

**Figure 2.**
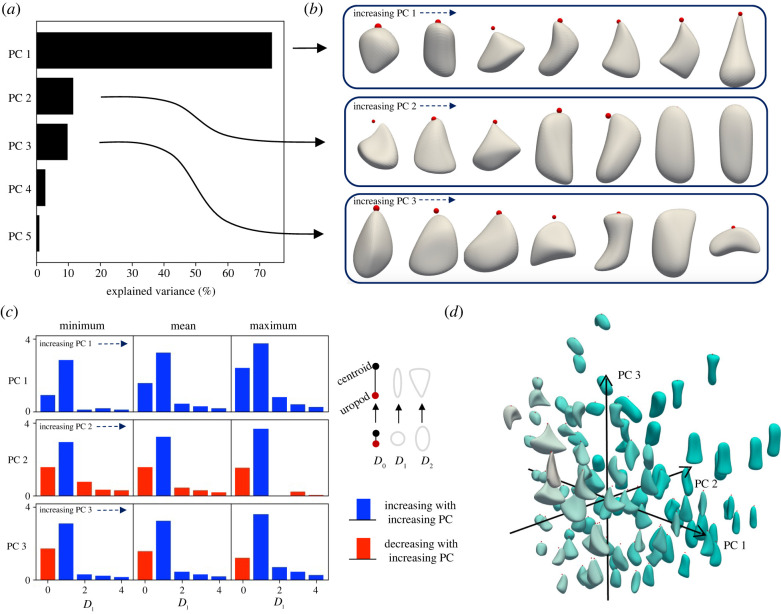
T cell shape is low-dimensional as quantified with three principal components. (*a*) Principal components (PCs) 1, 2 and 3 capture 74%, 12% and 9.8% (total of 96%) of the variance in *D*_*l*_, respectively. (*b*) Shape changes associated with each PC (*l*_max_ = 3 reconstructions), found by splitting the PCA space into seven equal-length bins along each axis and plotting the T cell within each bin with the lowest value for the other PCs. An increasing PC 1 represents elongation and front-widening, a decreasing PC 2 represents contraction with front-widening, and an increasing PC3 represents elongation (forward or sideways) with the centroid moving towards the uropod. (*c*) Correspondence between the principal components (PCs) and *D*_*l*_ is found by inverting the minimum, mean and maximum of each PC, with the other two PCs set to zero. Red and blue indicate decreasing and increasing descriptors, respectively, as the PCs are increased. *D*_0_ represents the closeness between the uropod and centroid, *D*_1_ the ellipsoidal aspects, and higher descriptors represent higher-frequency shape features. (*d*) Cell reconstructions with *l*_max_ = 3 at their positions in PCA space. Darkness of colour indicates increasing PC 2.

We used principal component analysis (PCA) to identify a set of uncorrelated linear features, or principal components (PCs), from the initial high-dimensional shape representation, {*D*_*l*_}. Despite the lack of obvious constraints from manual inspection, figure 2*a* shows that only three PCs are required to capture approximately 96% of the variance in the data (74%, 12% and 9.8% for PCs 1, 2 and 3, respectively). The rotational invariance means that the PCA coordinates are not invertible to unique shapes. To better isolate what features each PC describes, we therefore split the PCA space into 7 equal-length bins along each axis and plotted the T cell within each bin with the lowest value for the other PCs, shown in figure 2*b* for *l*_max_ = 3 reconstructions and electronic supplementary material, figure S2*a* for full cells (and electronic supplementary material, figure S2*b* shows the PC values of these plotted cells). Figure 2*c* shows what *D*_*l*_ transitions these PCs correspond to, with the minimum, mean and maximum inverted for each PC (with the other PCs set to zero), and electronic supplementary material, figure S2*c* shows the vector composition of each PC. An increasing PC 1 represents elongation and front-widening, a decreasing PC 2 represents contraction with front-widening, and an increasing PC3 represents elongation (forward or sideways) with the centroid moving towards the uropod. Figure 2*d* shows a sample of cells (with *l*_max_ = 3) at their locations in the PC space. Electronic supplementary material, figure S2*d* shows that along the main axis of variation (PC 1), dimensionality is relatively constant, and electronic supplementary material, figure S2*e* shows only modest differences in the spherical harmonic spectra of the low and high PC 1 populations.

Uncertainty in the uropod label, a diffuse region rather than a precisely locatable point, can be quantified and propagated to downstream variables of interest (electronic supplementary material, figure S3 and §4). Uropod uncertainty was found using the curvature around the labelled point (electronic supplementary material, figure S3*a*,*b*), and then PC uncertainties were calculated by re-computing *D*_0_ using each point on the cell rear within this uncertainty (electronic supplementary material, figure S3*c*). The mean per cent uncertainty in *D*_0_ is 1.5%, which is lower than the uropod uncertainty since cell rears are typically perpendicular to the axis defined by the centroid and uropod. The percentage uncertainties of the PCs (relative to their standard deviations) are 4.4%, 0.3% and 9.2% for PCs 1–3, respectively.

### 2.2. Run-and-stop migration emerges over long timescale

**Figure 3.**
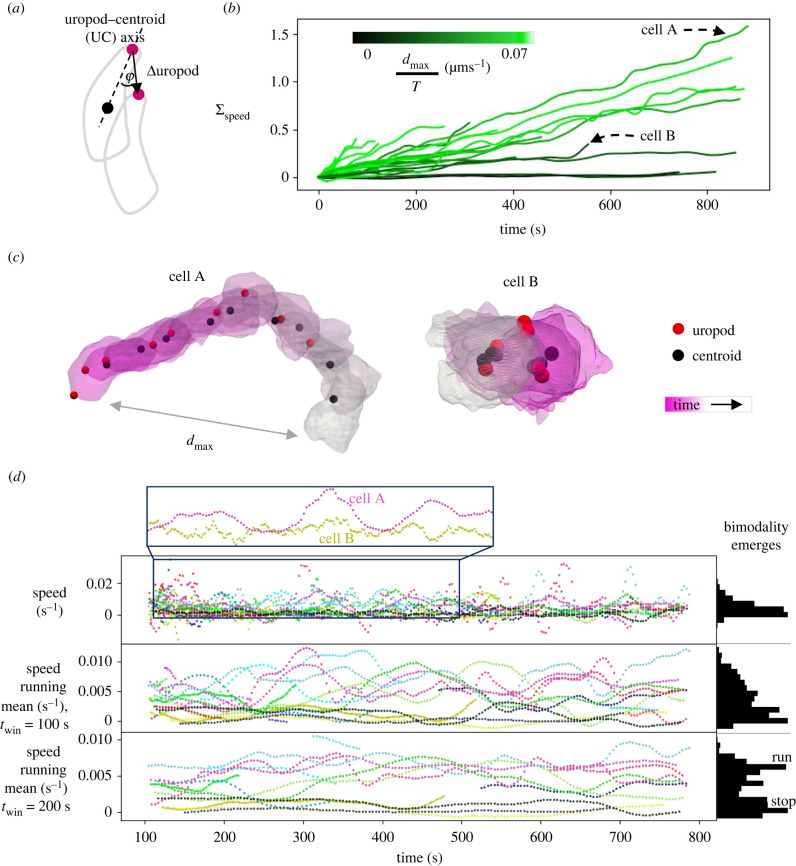
Run-and-stop migration emerges over long timescales. (*a*) Speed is defined as the uropod speed along the uropod–centroid (UC) axis, ||(Δuropod/Δ*t*)||cos*ϕ*, with smoothed uropod and centroid locations, and a further operation for invariance to cell scale (see §4). (*b*) Cumulative speed plots show that some cells have repeated phases of high speed (e.g. cell A), while others have much lower speeds (e.g. cell B). Lines are coloured by the maximum distance travelled divided by total dataset duration. (*c*) These speed differences are significant despite substantial uropod motion in some cases. Meshes are shown every approximately 104 s and 103 s for Cells A and B, respectively. (*d*) Histograms of speed with different running mean windows (*t*_win_). At small timescales, differences in speed between cells can be indistinguishable because most exhibit phases of low speed, highlighted for cells A and B. However, bimodality into two modes (run-and-stop) emerges at around 150 s.

To connect morphodynamics with migration strategies, variables describing cell motion are required. There are two landmark-like features of the cell that move through the ECM, the uropod and the centroid, and we calculated velocity vectors for both, invariant to cell scale (i.e. units of s^−1^; see §4). To ensure that uropod velocities have adequate signal-to-noise ratio (SNR), where noise arises from uropod labelling uncertainty, we found for each dataset the mean time taken for the uropod to move a significant distance, *τ*_sig_, and then computed velocities using running means over position with a time window of *τ*_sig_ (see §4 and electronic supplementary material, figure S3*d* for details). We then calculated speeds, which are one dimension and rotationally invariant, unlike velocities. The uropod and centroid speeds alone cannot separate distinct behaviours at small timescales, like translation and rotation (electronic supplementary material, figure S4*a*), and so we searched for a biologically meaningful reference frame. We found that long-timescale migration is typically along the axis defined by the uropod and centroid (the UC axis), rather than the ellipsoid major axis (electronic supplementary material, figure S4*b*). The speeds of the uropod and centroid along this axis then better differentiate distinct motifs (electronic supplementary material, figure S4*c*) and electronic supplementary material, figure S4*d* shows that these describe largely irreversible motion. The former has lower variance and fewer reversals (electronic supplementary material, figure S4*d*), and figure 1*f* and electronic supplementary material, video S9 show dynamics that we observed in some datasets, where the cell appears to test routes with multiple extensions and retractions but a relatively static uropod, before committing with the uropod. We therefore selected uropod speed along the UC axis as the variable for cell motion (figure 3*a*), and henceforth refer to it simply as speed.

Two migration modes separate out at long timescales, as shown in plots of cumulative speed (figure 3*b*): repeated phases of high speed, making significant progress forward, e.g. cell A; and lower speeds, yielding little progress, despite significant uropod motion in some cases, e.g. cell B (figure 3*c*). Figure 3*d* shows that, while at small times the dynamics can be indistinguishable (both modes have phases of near-zero speed), run-and-stop bimodality emerges at approximately 150 s. This bimodality is consistent with conclusions from lower-resolution experiments, where long-timescale trajectories of single cells have been modelled with Lévy-type random walks (characteristic of switching between stop and run modes) [29]. Interestingly, another study suggested more complex statistics, with cells divided into sub-populations described by distinct random walk models [30]. PCs 1 and 2 have a stronger correlation with run-and-stop mode than speed, indicating that shape is specialized more for migration mode than instantaneous speed, with cells in the run mode longer and thinner than those in the stop mode (electronic supplementary material, figure S5*a*). We next explored the morphodynamics behind these migration modes.

### 2.3. Stereotyped morphodynamics underlie migration modes

We analysed longer duration datasets for each of the run and stop modes to investigate how they differ (electronic supplementary material, videos S1–4 and 5–8 for the run and stop modes, respectively). We first computed the autocorrelation functions (ACF) of the shape (PCs 1–3) and speed dynamics (using high SNR timeseries; see §4 for details). The ACF is the correlation of a timeseries with a lagged version of itself, as a function of the lag, which can reveal the presence of latent variables preserving information across time. We found an autocorrelation decay time, *τ*_ACF_, by fitting an exponential decay model to the peaks of the oscillating ACFs (electronic supplementary material, figure S5*b*), and these decay times are indicative of the timescales over which processes are likely guided more by internal cytoskeletal machinery than stochastic external cues. For the stop mode, PC 3 is more autocorrelated than the other variables (mean *τ*_ACF_ ∼ 250 s compared with approx. 150 s of the other variables; electronic supplementary material, figure S5*b*). PC 3 dynamics are suggestive of sensing: forward extension with a tentative rearward centroid, and reaching sideways. See electronic supplementary material, videos S5–9, with the three included in the PC 3 ACF analysis coloured by PC 3. For the run mode, the main differences are a decrease in the PC 3 autocorrelation (to approx. 150 s) and an increase in the speed and PC 2 (contraction with front-widening) autocorrelations (to approx. 225 s). The power spectra in electronic supplementary material, figure S5*c* show that the run mode has larger oscillations in speed and PC 2, particularly for 0.005–0.01 Hz. The run mode is therefore associated with faster oscillations in speed and PC 2 that typically remain autocorrelated for longer than those of the stop mode. These ACFs give a global perspective on morphodynamics, and the presence of long timescales suggests that the morphodynamics are, as with the morphologies, low-dimensional. We therefore next zoomed in on the PC timeseries to interpret the organization of local morphodynamics, or ‘behaviours’, that underlies this low-dimensionality.

**Figure 4.**
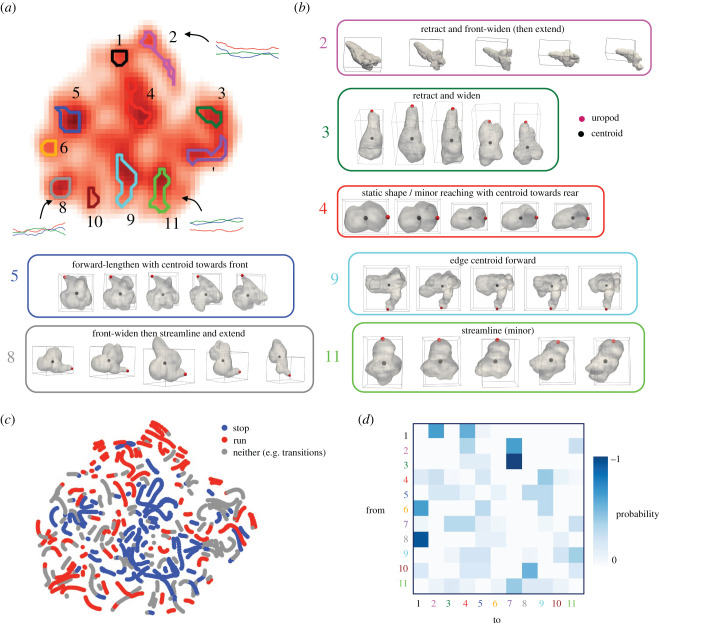
Morphodynamics are organized in stereotyped motifs. (*a*) Eleven stereotyped motifs are local peaks in a probability density function (PDF) over the spectrogram embeddings in morphodynamic space, where different locations represent different local morphodynamics. These form more of a discrete set than continuum, and some examples of the local PC series are shown (red, blue and green lines for PCs 1–3, respectively). (*b*) Example stereotyped motifs, with frames evenly spaced across 150 s. (*c*) Utilization of motifs in the stop and run modes. Red and blue indicate that the speed running mean is above 0.005 s^−1^ (run mode) and below 0.0025 s^−1^ (stop mode), respectively (selected from the bimodal distribution in figure 3*d*), and grey indicates it is in between these values (e.g. transitions). (*d*) Transition probability matrices reveal how cells move around the morphodynamic space, counting transitions only once the cell moves to a different motif.

The continuous wavelet transform is a method for finding local morphodynamics (behaviours) from a timeseries of morphologies, and has been used to map stereotyped behaviour in fruit flies [23,24]. See §4 for the full pipeline and electronic supplementary material, figure S6*a* for a schematic. Wavelets are used to transform the timeseries into a spectrogram with multiscale dynamic information. Dimensionality reduction with *t*-SNE [31] can then be performed to map the spectrogram to an interpretable two-dimensional morphodynamic space, where different locations represent different local morphodynamics (figure 4*a*), and electronic supplementary material, figure S6*b* shows the dimensionality reduction is robust across different hyperparameters. Stereotyped motifs are those that are frequently performed, and so correspond to peaks in the probability density function (PDF) of spectrogram embeddings in this space. We used wavelets with a maximum width of influence of 150 s, the approximate timescale of organization found from the autocorrelation analysis.

We found that behaviours are organized into more of a discrete set rather than a continuum (figure 4*a*), forming ‘islands’ between which cells jump, and we could therefore categorize and interpret these individually. Figure 4*b* shows key examples, with frames evenly spaced over a 150 s interval (with the remainder and further examples in electronic supplementary material, figure S7), and electronic supplementary material, figure S8 shows the PC dynamics of three examples from each motif. Figure 4*c* shows how these are used differently in the run and stop modes. Red and blue indicate that the speed running mean is above 0.005 s^−1^ (run mode) and below 0.0025 s^−1^ (stop mode), respectively (selected from the bimodal distribution in figure 3*d*), and grey indicates it is in between these values (e.g. transitions). In the stop mode, stereotyped motifs include static shape or minor reaching with centroid towards the rear (4); forward lengthening with centroid towards the front (5); and edging centroid forward (9) (figure 4*b*). In the run mode, stereotyped motifs include front-widening then streamlining and extending (8); and retracting and front-widening then extending (2). A probability matrix for transitions between the stereotyped motifs is shown in figure 4*d*, with rows and columns corresponding to the start and end motifs, respectively. We assigned points to the closest stereotyped motif and counted transitions only once the cell moves to a different motif (i.e. diagonal entries are zero). Frequent transitions include from 3 to 7 (retract to reach to one side) and from 8 to 1 (front-widen then streamline and extend to front-widening).

We next looked in detail at the run mode, of particular interest as this is when cells use global morphodynamics for active translocation through the ECM, and because it is in all cases defined by polarized morphologies, for which our descriptor was designed. First, we repeated the wavelet analysis with the longer duration datasets of the run mode, finding more of a continuum than the global morphodynamic space, but for which stereotyped motifs can still be categorized (electronic supplementary material, figure S9*a*,*b*, with PC timeseries of three examples from each motif in electronic supplementary material, figure S10). Electronic supplementary material, figure S9*c*,*d* shows the speeds and transition probability matrix. Apart from a turning motif, all fall into two categories: compression, and a rearward surface motion with extension forward (rearward with respect to the cell frame of reference, and relatively static in the laboratory frame). The precise motifs are then variants on these base behaviours, e.g. whether there is also widening. These underlying morphodynamics, omitting the distracting variations, are most characteristic of PC 2 dynamics, and this connection between PC 2 dynamics and migration is certainly consistent with the increased autocorrelation timescales and power of PC 2 relative to the stop mode (electronic supplementary material, figure S5*b*,*c*).

**Figure 5.**
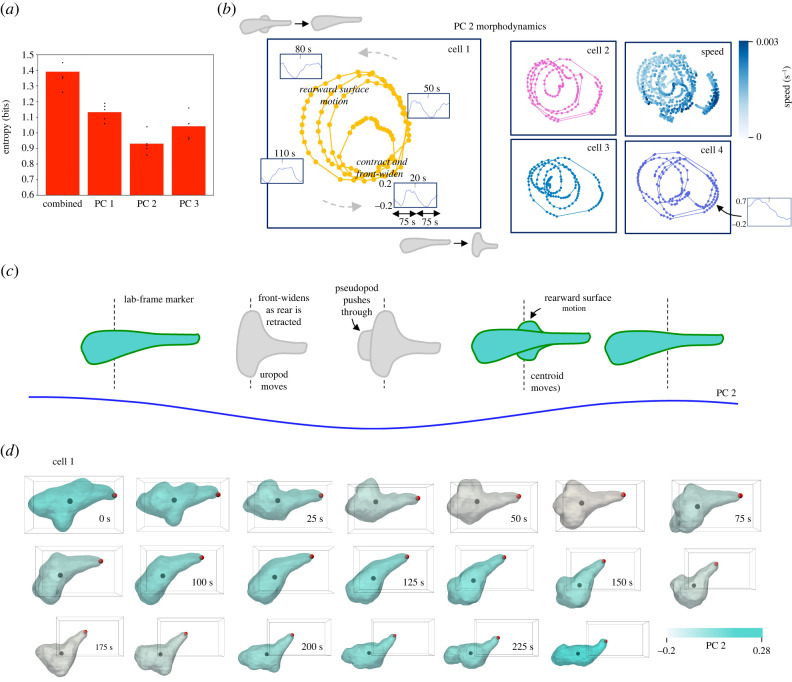
Periodic oscillations in PC 2 underlie the run mode. (*a*) Entropy of the run mode marginal dynamics for each PC (5 repeats for each) shows a minimum for PC 2, and therefore that these dynamics are the most stereotyped, consistent with the autocorrelation results of electronic supplementary material, figure S5*b*. Markov chain entropies were calculated for transitions on grids over morphodynamic spaces for each PC, found by repeating the wavelet analysis with each PC on its own. (*b*) Dynamics in the PC 2 morphodynamic space for the run mode, where different locations represent different local PC 2 morphodynamics (and decreasing PC 2 represents contraction and front-widening). Tracking the trajectories of the longer duration datasets reveals periodic oscillations of varying amplitude. Local PC 2 dynamics in 150 s windows are shown inset at key points in the morphodynamic space, showing that outer rings represent higher-amplitude oscillations, with a region for particularly large PC 2 decreases, bottom right. The top left corner represents rearward surface motion and the bottom right corner represents contraction and front-widening. Regions in between represent transitions between these motifs. Maximum uropod speeds correspond to contraction and front-widening. (*c*) These results are suggestive of the following cyclic morphodynamic propulsion mechanism: the leading edge widens, likely intercalating with the ECM and contracting the uropod; the leading edge then extends forward, as the previously widened leading edge regions undergo a rearward motion that may connect with the ECM like a paddle. This cycle repeats every approx. 100 s. (*d*) An example showing these oscillations in cell 1, coloured by PC 2.

To test this theory, we calculated the entropy of each PC’s morphodynamics. We did this by repeating the wavelet analysis for each PC on its own and calculating the Markov chain entropy for transitions on a grid over the resulting morphodynamic space (see §4 and electronic supplementary material, figure S9*e*). We used grids since these dynamics formed continuums rather than discrete, categorizable morphodynamic spaces. We found an entropy minimum for PC 2 (figure 5*a*), confirming that PC 2 dynamics are the most stereotyped. Figure 5*b* shows how all four cells follow the same circular oscillations of varying radius in the space of PC 2 morphodynamics. Electronic supplementary material, videos S1–4 are labelled as cells 1–4. Outer and inner rings represent high- and low-amplitude oscillations, respectively, and there is a region for particularly large decreases in PC 2 (but not for large increases). These results, in conjunction with electronic supplementary material, videos S1–4, coloured by PC 2, suggest the following morphodynamic propulsion mechanism (sketched in figure 5*b*,*c*): the leading edge widens, likely intercalating with the ECM to contract the uropod (PC 2 decreases); the leading edge then extends forward, as the previously widened leading edge regions undergo a rearward flow that may connect with the ECM like a paddle, ultimately streamlining (PC 2 increases). This cycle is repeated every approx. 100 s, and explains the oscillations in (uropod) speed observed in figure 3*d*. Figure 5*d* shows an example section of electronic supplementary material, video S1, coloured by PC 2. These results suggest that T cells use a highly periodic internal machinery to generate a sustained migration effort, alternating between two previously proposed propulsion mechanisms to move the uropod then leading edge forward [7,9,10]. A plausible mechanistic basis for the rearward morphodynamic flow is retrograde cortical actin flow, a process that has been implicated in amoeboid migration in a number of cells, including T cells [9,10]. However, further investigations of internal actin dynamics are needed to explore this connection.

## 3. Discussion

T cells are a key part of the adaptive immune system, migrating through the ECM to neutralize infected and cancerous cells. However, their morphodynamics have not yet been completely quantitatively mapped in three dimensions. Here, we used LLSM to acquire datasets of primary mouse cytotoxic T cells migrating through a collagen matrix with high spatio-temporal resolution. Using a novel shape descriptor that incorporates key polarization information with a uropod label, we found that shape was low-dimensional. Run-and-stop migration emerges at long timescales (approx. 150 s), and global morphodynamics are stereotyped, forming a discrete set rather than continuum. Stop mode morphodynamics primarily involve oscillations in centroid movement towards the uropod, with extension forwards or sideways (PC 3 dynamics), and these remain autocorrelated for long timescales (decay time, *τ*_ACF_ ∼ 250 s). The run mode (i.e. active translocation) arises from periodic oscillations in PC 2, with a period of approximately 100 s and *τ*_ACF_ ∼ 225 s: the leading edge widens, likely using intercalation with the ECM to contract the uropod (PC 2 decreases); the leading edge then extends forward, as the previously widened leading edge regions undergo a rearward motion that may connect with the ECM like a paddle, ultimately streamlining (PC 2 increases). These results indicate that periodicity in the cellular machinery helps to sustain forward motion during active translocation.

Uropod tracking proved vital for differentiating key morphological and morphodynamic states. Uropod uncertainties were then required to ensure that analysis was at sufficient SNR, because the uropod is a diffuse region rather than a precisely locatable point. In analogy to the role of the Hessian matrix in parameter fitting, we found this could be achieved relatively simply by quantifying uropod uncertainty through the curvature of the cell rear, then propagating this to downstream variables of interest. The inclusion of landmark-like but diffuse features will likely become more important as methods for tracking intracellular structures at high spatio-temporal resolution continue to improve, meaning spatial regions can be associated with specific internal organization and activity [32]. In a small number of cases (e.g. electronic supplementary material, video S7), thin fluid-like protrusions extend out of the uropod, which cause dynamics in *D*_0_ that are unlikely to be important for migration. To reduce these effects, in future work, we will explore labelling uropods based on smoothed reconstructions (with e.g. *l*_max_ = 15). We found uropod definition reduced for some cells in a long-lived stop mode (and therefore had high uncertainties for some PCs, meaning they were omitted from analyses). This may be indicative of loss of polarization, so for these modes alternative shape descriptors may be more appropriate.

Internal retrograde actin flow has been a hallmark of cell migration models for decades, since Abercrombie first observed centripetal flow of particles on fibroblast surfaces [5,10]. However, Abercrombie also proposed a second propulsion mechanism, where rearward flows of surface deformation might push the cell forward like a paddle. Such morphodynamic flows (or ‘waves’) have recently been observed in two-dimensional migrating *Dictyostelium* cells [33], and in T cells embedded in microfluidic channels where they can enable migration without any adhesion [9]. To our knowledge, however, they have not been characterized in three-dimensional ECM environments. Through inhibition at obstacles and activation on the opposite side, flows may also aid turning as has been described in neutrophils [34], and the lateral protrusions likely serve as an anchor in confined geometries [35,36]. Analysis of actomyosin dynamics, as well as tracking of the ECM fibres (perhaps with a contact map over the cell surfaces), would help test the connection between the rearward surface motion and internal actin dynamics, and the specific nature of how these interact with the ECM for anchoring and propulsion. The analysis would also reveal the extent to which decreasing PC 2 (contraction with front-widening) is driven by contact with fibres, although the periodic PC 2 dynamics across all run mode cells suggests this may predominantly be internally regulated.

Exciting areas for future work include the extension of the analysis to the timescale of hours, where the statistics and morphodynamics of switching between run and stop modes could be interpreted at the single-cell level, and the hierarchical organization of the stereotyped motifs could be mapped. There are technical challenges, however: individual cells would have to be followed and migration distances would exceed the scales of current LLSM fields of view. Dataset sizes might also become problematic, given that a 20 min video corresponds to 1 TB of data (with one colour). Furthermore, non-stationary issues such as ageing, differentiation and activation may come into effect [37]. It would also be interesting to build statistical models of T cell morphodynamics [16], which may then enable the development of mechanistic models [38], connecting morphodynamics to both extracellular and intracellular processes.

Ultimately, we hope quantitative morphodynamic analyses of T cells navigating the complex ECM environment will aid comparison of migration across different conditions (e.g. tissues, drugs and cell mutants). In particular, the prevalence and switching between human-labelled modes of migration such as chimneying, mesenchymal, amoeboid (blebbing), finger-like and rear-squeezing could be put on firm objective grounds [38,39]. These advanced morphodynamic analyses will in turn help the development of mechanistic models, with a view to enhanced understanding of, and more effective, immunotherapeutics.

## 4. Methods

### 4.1. Lattice light-sheet microscopy imaging and pre-processing

#### 4.1.1. Lattice light-sheet microscopy

LLSM experiments were either performed on a custom-built system described in [40], or on a Zeiss Lattice Light Sheet 7 microscope (Zeiss, Oberkochen, Germany). OT1-Lifeact-GFP T cells [41] labelled with CellTracker Deep Red dye were excited at 642 nm, OT1-mT/mG at 561 nm and plain OT1-Lifeact-GFP T cells at 488 nm. For all experiments performed on the home-built system, Point Spread Functions (PSFs) were measured using 200 nm Tetraspeck beads. The acquired datasets were deskewed and deconvolved using LLSpy, a Python interface for processing of LLSM data. Deconvolution was performed using a Richardson–Lucy algorithm using the PSFs generated for each excitation wavelength. Datasets acquired on the Zeiss system were deskewed using the Zeiss Zen (blue edition) software. All data were acquired at 37°C and 5% humidified CO_2_. The voxel size was 0.1 × 01 × 0.2 μm^3^ for the home-built system and 0.145 × 0.145 × 0.4 μm^3^ for the Zeiss system. The temporal resolution was 2.5 s per frame for the OT1-mT/mG datasets, 5.6 s per frame for the OT1-Lifeact-GFP-CellTracker Deep Red datasets (both imaged on the home-built system) and 4.17 s per frame for the plain OT1-Lifeact-GFP datasets imaged on the Zeiss system. We collected 29 datasets with 2850 frames altogether and a mean and standard deviation across datasets of 98 and 78, respectively.

#### 4.1.2. Image segmentation

Before further processing, membrane Tomato signal was denoised using a deep-learning approach based on Content-Aware Image Reconstruction (CARE) [42]. CellTracker Deep Red and membrane Tomato signal were bleach-corrected using Fiji [43]. Cell surfaces were segmented using Imaris 8.4.1 (Bitplane, Zurich, Switzerland). To minimize the occurrence of holes in the surfaces, depending on the signal to noise, ratio smoothing factors between 0.35 μm and 0.8 μm were applied. Cell surface triangulations were exported using custom Matlab code and again analysed for surface holes. If required, surface holes were eliminated by custom Matlab code based on closing operations.

#### 4.1.3. Sample preparation

Primary murine OT1-Lifeact-GFP and OT1-mT/mG cytotoxic T cells were isolated and cultured as previously described [41]. All imaging was done with T cells cultured over 6 or 7 days. For imaging on the home-built system, OT1-Lifeact-GFP T cells were labelled with 100 nM CellTracker Deep Red dye (ThermoFisher Scientific, Waltham, MA, USA). Keeping all components on ice, collagen matrix solution was prepared by adding 10 μl of 10 × PBS, 1.15 μl 1N NaOH and 39 μl T cell medium (TCM), consisting of phenol-free RPMI 1640, 10% foetal calf serum, 1 mM sodium pyruvate, 10 mM HEPES, 100 U ml penicillin, 100 μg ml streptomycin, 2 mM *L*-glutamine and 50 μM *β*2-mercaptoethanol (all from Gibco, ThermoFisher Scientific), to 50 μl liquid-phase rat-tail collagen I (approx. 3 mg/ml; Corning, New York, NY, USA). Coverslip and imaging dish glass surfaces were treated with 2% (3-aminopropyl) triethoxysilane in ethanol and 6% glutaraldehyde to facilitate firm attachment of collagen gels. For imaging on the home-built LLSM, 6 μl of collagen mix were placed onto surface-treated round 5 mm coverslips (Warner Instruments, Hamden, CT, USA) and polymerized at 37°C for 15 min. After polymerization, 10^5^ T cells in phenol-free TCM were seeded on top of the gel and allowed to infiltrate over 3 h before imaging. For imaging on the Zeiss LLS system, 10^5^ T cells were added to TCM during collagen matrix mix preparation. Seventy millilitres of collagen mix were added to well of 35 mm imaging dishes (Mattek, Ashland, USA) and polymerized at 37°C for 30 min. After polymerization, 1 ml of pre-warmed phenol-free TCM was added to the dish and cells were allowed to recover for 1 h before imaging.

### 4.2. Quantifying three-dimensional cell morphology

Cell morphologies were quantified using SPHARM. First, the cell surface, described with three Cartesian coordinates, {*x*, *y*, *z*}, is mapped to the unit sphere, described with polar coordinates {*θ*, *ϕ*}, such that the three Cartesian coordinates are functions of the polar coordinates: {*x*(*θ*, *ϕ*), *y*(*θ*, *ϕ*), *z*(*θ*, *ϕ*)}. {*x*(*θ*, *ϕ*), *y*(*θ*, *ϕ*), *z*(*θ*, *ϕ*)} are then be decomposed in terms of the spherical harmonics, $Y_{l}^{m}(\theta ,\phi )$, and only *m* ≥ 0 functions are required [44]. For *x* for example,

4.1 $$x(\theta ,\phi )=\sum_{l=0}^{\infty }\sum_{m=0}^{l}c_{l,x}^{m}Y_{l}^{m}(\theta ,\phi ),$$

and the (in general complex) coefficients, $c_{l,i}^{m}$ with *i* ∈ {*x*, *y*, *z*}, represent the morphology. We used the Spharm-pdm software package [44] to find the coefficients for the T cells with *l*_max_ = 15 and cell meshes converted to voxel grids with a spatial resolution of 0.5 μm for computational speed. The additional variable for capturing polarization information was *D*_0_. This was the distance between the uropod and centroid multiplied by $\frac{3}{2}$, with the numerator reflecting the fact that the harmonics are summed over three spatial coordinates and the denominator accounting for the fact that the coefficients have a spatial extent double their magnitude. The uropod was manually selected (aiming for its centre) in alternating frames and linearly interpolated. PCA is a dimensionality reduction method that finds a set of uncorrelated linear features (the PCs), which are the eigenvectors of the data covariance matrix (which for *D*_*l*_ has dimensions 16 × 16) [45]. Electronic supplementary material, figure S2*c* shows the vector composition of each PC. As explored through the main text, PC 1 is largely associated with transitions between run and stop mode morphologies, PC 2 is largely associated with morphological transitions in the run mode, and PC 3 is largely associated with morphological transitions in the stop mode. For implementing PCA, we used the Scikit-learn Python package.

### 4.3. Uncertainty quantification

The uncertainty in the uropod label depends on the curvature of the cell rear, which we quantified using the mean curvature averaged across the 15 closest mesh vertices to the labelled point (with a sub-sampled mesh for computational speed). We then defined the positional uncertainty as the cord length associated with a 20° rotational perturbation. To convert this to PC uncertainties, we found the set of possible *D*_0_ values using mesh vertices within this uncertainty (i.e. within one cord length of the uropod label), calculated the standard deviation, and converted to PC uncertainties by multiplying by the cosine of the angle between the *D*_0_ and PC vectors in {*D*_*l*_} space. This process was repeated for every 10 frames in each dataset to get a single characteristic uncertainty for each PC (the mean) for each dataset. *τ*_sig_ was calculated as the mean time taken for the uropod to move twice the cord length. Some cells have uropods that are near-stationary, and therefore have a *τ*_sig_ comparable with the full dataset duration. To account for such cases, we used a maximum *τ*_sig_ of 100 s, in order that these could be plotted for comparison with dynamic cells, but we excluded them from quantitative analysis.

### 4.4. Finding a motion variable for small timescales

We calculated uropod and centroid velocities by finding the displacements between consecutive positions (smoothed with running means over *τ*_sig_ for both, for consistency) and dividing by the time step and cube root of cell volume (for invariance to cell scale). The ellipsoid major axis was calculated as the eigenvector with the largest eigenvalue of *A*^*T*^*A*, where A is a matrix of the *l* = 1 spherical harmonic coefficients [25]: $\sqrt{3}/(3\sqrt{2\pi })(c_{1}^{-1}-c_{1}^{1},i(c_{1}^{-1}-c_{1}^{1}),\sqrt{2}c_{1}^{0})$. For comparing the uropod-centroid (UC) and ellipsoid axes, we used running means for uropod and centroid with a time window of 100 s for long-time-scale behaviour. We compared cells where the uropod speed was above 0.0025 s^−1^, i.e. moving more than a quarter cell length in 100 s, and the distance between the uropod and centroid velocity vectors was within half the uropod speed, i.e. they were aligned.

### 4.5. Time-series autocorrelation functions and power spectra

ACF and power spectra were computed for longer duration datasets for each of the run and stop mode. We removed timeseries with low SNR: PC timeseries where the ratio of the signal standard deviation to the PC uncertainty was below 2.5 and speed timeseries where *t*_sig_ was of a similar scale to the full dataset duration. There was one removal for each of PC 1, 3 and speed, across different cells. We calculated the autocorrelation on de-trended timeseries, in order to only capture statistically significant correlations, removing trends with frequencies lower than 0.0025 Hz (corresponding to a period of approximately half the total dataset duration) with a Butterworth high-pass filter [46]. We then found a decay time, *τ*_ACF_, by fitting an exponential decay model, $y=e^{-(x/\tau_{\mathrm{ACF}})}$, to the peaks of the ACF (rather than the full ACF, which is more appropriate for non-oscillatory patterns).

### 4.6. Continuous wavelet transform

The continuous wavelet transform was used to find local morphodynamics (or ‘behaviours’) from the PC timeseries. A wavelet that decays to zero on either side of a central peak is convolved with the timeseries, which produces a new timeseries where each element now represents local morphodynamics. Repeating this process with dilated versions of the wavelet and stacking the resulting set of timeseries yields a spectrogram with multiscale dynamic information, where high-frequency components are analysed close in time, but lower frequency information bleeds in from afar. This spectrogram is then mapped to an interpretable two-dimensional space using *t*-SNE (*t*-distributed stochastic neighbour embedding) [31], and a PDF can be computed with kernel density estimation [47]. *t*-SNE is a nonlinear dimensionality reduction method that uses machine learning. Two similarity metrics between datapoints are defined for each of the two representations, the initial (high-dimensional) representation and the target (lower-dimensional) representation. The difference between the distributions of these similarities across all data pairs is minimized. For implementing *t*-SNE, we used the Scikit-learn Python package with default parameters: perplexity (analogous to the number of neighbours in other algorithms) of 30, learning rate of 200 and 1000 iterations.

We identified stereotyped motifs (PDF peaks) using adaptive binarization, a method that thresholds pixels in an image with a threshold value that depends on the local statistics: the mean over a surrounding square of pixels with an added bias (we used square dimensions of 7 and a bias of 20, found with a grid search). We used adaptive rather than pure binarization so that regions with high-density peaks and high PDF between then (‘superhighways’ representing common transitions) could be separated, while lower peaks in absolute terms could also be captured. We used two simple wavelets, the ‘Mexican hat’ wavelet and Gaussian first derivative wavelet, with the combination of the two required to capture symmetric and antisymmetric features. For organisms where the morphodynamics of interest are organized in repeating bouts, e.g. high-frequency wing-beating of fruit flies, complex wavelets that enable the removal of phase information can be useful. However, over the timescales analysed here, T cell morphodynamics are slower-changing, and phase information is important. We used six equally spaced frequencies for each wavelet from double the Nyquist limit up to the (wavelet-specific) frequency with width of influence corresponding to 150 s, the approximate timescale of organization found from the autocorrelation analysis. The width of influence was found by convolving each wavelet with a square pulse to find where edge effects begin. When repeating this method for only the four run mode datasets, we used for the adaptive binarization parameters square dimensions of 15 and a bias of 50, again found with a grid search.

### 4.7. Comparing marginal morphodynamics of the run mode

The marginal morphodynamics form continuums, and so transition matrices over stereotyped PDF peaks cannot be defined. Instead, we defined transition matrices over points on a grid. We then quantified the entropy for the transition dynamics of each PC (and compared with that of their combined dynamics). The entropy is $-\sum \pi_{i}p_{ij}\log_{2}p_{ij}$, where *π*_*i*_ is the equilibrium distribution and *p*_*ij*_ is the probability that the next motif to be visited after *i* will be *j*. For plotting the PC 2 dynamics of the four cells, we perturbed the wavelets slightly to further improve the interpretability. This was done by searching locally across options for the maximum wavelet width (keeping 150 s as an upper bound) and finding combinations with reduced entropy. Reduced entropy was associated with reducing the Gaussian wavelet maximum width to 100 s, but with the same Mexican hat wavelets as before.

## Data Availability

The cell surface segmentation data that support the findings of this study are available from the Dryad Digital Repository: https://doi.org/10.5061/dryad.tdz08kq1r [48]. The code used is available at https://github.com/hcbiophys/tcells_paper_code.
